# Development of Aggression Subtypes from Childhood to Adolescence: a Group-Based Multi-Trajectory Modelling Perspective

**DOI:** 10.1007/s10802-018-0488-5

**Published:** 2018-11-07

**Authors:** Lisa-Christine Girard, Richard E. Tremblay, Daniel Nagin, Sylvana M. Côté

**Affiliations:** 1 School of Health in Social Science, Clinical Psychology, Medical School, University of Edinburgh, Teviot Place, Edinburgh, EH8 9AG UK; 2 School of Public Health, Physiotherapy, and Sports Science, University College Dublin, Dublin, Ireland; 3 Research Unit on Children’s Psychosocial Maladjustment (GRIP), Université de Montreal, Montréal, Canada; 4 Departments of Pediatrics and Psychology, Université de Montreal, Montréal, Canada; 5 Heinz College, Carnegie Mellon University, Pittsburgh, PA USA; 6 Bordeaux Population Health, Inserm Research Centre for Epidemiology and Biostatics, U1219 team Healthy, Université de Bordeaux, Bordeaux, France

**Keywords:** Aggression, Developmental trajectories, Group-based modelling, Longitudinal

## Abstract

The persistence of elevated subtypes of aggression beginning in childhood have been associated with long-term maladaptive outcomes. Yet it remains unclear to what extent there are clusters of individuals following similar developmental trajectories across forms (i.e., physical and indirect) and functions (i.e., proactive and reactive) of aggression. We aimed to identify groups of children with distinct profiles of the joint development of forms and functions of aggression and to identify risk factors for group membership. A sample of 787 children was followed from birth to adolescence. Parent and teacher reports, and standardised assessments were used to measure two forms and two functions of aggressive behaviour, between six and 13 years of age along with preceding child, maternal, and family-level risk-factors. Analyses were conducted using a group-based multi-trajectory modelling approach. Five trajectory groups emerged: non-aggressors, low-stable, moderate-engagers, high-desisting, and high-chronic. Coercive parenting increased membership risk in the moderate-engagers and high-chronic groups. Lower maternal IQ increased membership risk in both high-desisting and high-chronic groups, whereas maternal depression increased membership risk in the high-desisting group only. Never being breastfed increased membership risk in the moderate-engagers group. Boys were at greater risk for belonging to groups displaying elevated aggression. Individuals with chronic aggression problems use all subtypes of aggression. Risk factors suggest that prevention programs should start early in life and target mothers with lower IQ. Strategies to deal with maternal depression and enhance positive parenting while replacing coercive parenting tactics should be highlighted in programming efforts.

**Electronic supplementary material:**

The online version of this article (10.1007/s10802-018-0488-5) contains supplementary material, which is available to authorized users.

## ᅟ

Much attention has been paid to the onset and developmental course of different forms and functions of aggression throughout the lifespan. Rightly so given the individual, societal, and economic burden of aggression that persists beyond the normative period during early childhood. Research suggests that chronic aggression of one type or another is associated with many negative life outcomes. For example, chronic physical aggression during childhood has been linked to violent delinquency, criminality, and stable unemployment into adulthood (Broidy et al. 2003; Kokko and Pulkkinen 2000; Nagin and Tremblay 1999). Indirect aggression, which is characterised by social behaviours that are often covert in nature and used to manipulate others within a social context, has been associated with positive ‘reinforcing’ shorter-term outcomes such as being ‘*perceived popular*’ (Cillessen and Rose 2005; Hawley 2003) and negative outcomes such as internalizing symptoms, depression, suicide ideation, and substance abuse (Herrenkohl et al. 2009; Murray-Close et al. 2007; Van der Wal et al. 2003).

Within the early childhood perspective of aggression, research has shown that physical aggression commences as early as infancy, when the capacity to inflict harm onto others, such as hitting, kicking, and biting develops (e.g., Tremblay et al. 1999; Vitaro et al. 2006; Tremblay et al. 2018), coinciding with the development of infant’s early motor ability. The early childhood perspective of aggression emerged with the increasing use of longitudinal cohort studies, which shed a different developmental perspective to the previously held traditional theoretical framework of learned aggression resulting in the increased use of physical aggression as children age (e.g., Bandura 1973; Loeber and Stouthamer-Loeber 1998). Direct observations from longitudinal cohort studies of early development supported the perspective that engagement in physical aggression is instead at its peak during infancy and early childhood, in particular during the toddler years (Hay et al. 2014), with less frequent engagement in physical aggression as children develop (Broidy et al. 2003; Nagin and Tremblay 1999; Tremblay et al. 1996); rather than onset in late childhood or adolescence. This suggested the unlearning of an instinctual behaviour rather than a learned behaviour via observation and modeling. These observed decreases in the use of physical aggression following early childhood are argued to coincide with the increasing ability to self-regulate, coupled with children’s increasing cognitive and language development, and social information processing (Dodge and Frame 1982; Dodge and Schwartz 1997; Dionne et al. 2003; Séguin et al. 2009; Girard et al. 2014).

On the other hand, engagement in indirect forms of aggression, also observed in early childhood, has been found to increase in frequency as children develop from late childhood and into adolescence (Cairns et al. 1989; Vaillancourt et al. 2007). Börkqvist and colleagues’ developmental perspective suggested that these increases in indirect aggression coincide with the development of cognitive and language skills, alongside a better understanding of social norms and expectations (Björkqvist et al. 1992a; b; Björkqvist 1994). That is, the development of higher-order cognitive and language ability, coupled with a better understanding of social norms which would not support continual engagement in overt forms of aggression, would necessarily be expected to precede the use of a more sophisticated form of aggression such as indirect aggression. Thus, a developmental model of physical and indirect aggression would suggest early onset and higher frequency of physically aggressive behaviours in early childhood, which are then replaced by more covert forms of aggression, i.e., indirect aggression, as children develop in later childhood and adolescence (Björkqvist et al. 1992a; b; Tremblay et al. 1996). Taken together, independent engagement in either form of aggression has been shown to differ in both etiology and consequence, as well as present differently with respect to their developmental trajectories, despite the moderate-strong associations found between them (e.g., Crick et al. 2006; Kaukiainen et al. 1999; Vaillancourt et al. 2003; Côté et al. 2007).

### Functions of Aggression

While theoretical perspectives differentiate forms of aggression, functions of aggression are similarly differentiated. For example, proactive aggression, which is calculated, instrumental, and predatory in nature, has been linked to gang membership, substance abuse, delinquency, anti-sociality and psychopathic features in adulthood (e.g., Barker et al. 2006; Fite et al. 2010; Vitaro et al. 1998). Proactive aggression has been argued to coincide with a social learning model of aggression (Bandura 1973; Dodge and Coie 1987), in so much as the aggression can be operationalized as a learned behaviour that is goal driven. That is, the aggression is used to obtain an instrumental goal or reward (e.g., a desired object or social status within the peer group), and reinforced via operant conditioning (i.e., goal attainment). Within this framework, proactive aggression ought to either remain stable, or increase overtime.

Reactive aggression on the other hand, often provoked by anger in reaction to a perceived threat, is defensive in nature and has been associated with internalizing difficulties such as negative affect, depression, anxiety and additionally problems with self-regulation (Vitaro and Brendgen 2011). Reactive aggression has predominantly been operationalized within Berkowitz’s frustration model of aggression (Berkowitz 1988, 1989) given that the aggression is reactive rather than instrumental. It is a consequence of perceived provocation resulting in anger and retaliatory responses. Deficits in self-control, emotional regulation, and impulsivity are characteristics of high levels of reactive aggression (Denson et al. 2012; Marsee and Frick 2007). As a result, reactive aggression is likely to decrease with brain maturation across development and as children become better able to self-regulate (Tremblay 2000). While there was large debate surrounding the utility of distinguishing between reactive and proactive aggression, particularly given challenges of correct identification, studies continue to confirm their discriminative validity (e.g., Dodge and Coie 1987; Poulin and Boivin 2000; Kempes et al. 2005), along with distinct etiologies and consequences (e.g., Dodge 1991; Paquin et al. 2017; Vitaro et al. 2002).

## Trajectories of Forms and Functions of Aggression

Longitudinal studies from early childhood to adolescence have examined both single and joint trajectories of either forms, (i.e., physical and indirect; e.g., Cleverley et al. 2012; Côté et al. 2007) or functions of aggression (i.e., reactive and proactive; e.g., Barker et al. 2006), revealing heterogeneity in trajectories. Yet, to the best of our knowledge, no study to date has combined both forms and functions of aggression within a developmental perspective from childhood to adolescence, whilst examining their given co-occurring trajectories using a person-centered approach. This is an important next step given the distinction between forms and functions of aggression highlighted first by Pitkänen in Pitkänen 1969, and more recently by Little et al. (2003); coupled with the suggestion that even though functions are largely dependent on the form taken, proactive, reactive, physical and indirect aggression are nevertheless recognizably distinguishable constructs. This may help to better understand the prevalence of distinct yet co-occurring trajectories of multiple forms and functions of aggression from childhood to adolescence. This developmental period is particularly salient given social-cognitive-behavioural development that is occurring, which can facilitate either engagement or desistance in both forms and functions of aggression.

By taking into account the form of the aggressive behaviours (physical vs. indirect) as well as their function (proactive vs. reactive) within longitudinal studies, we are more likely to understand to what extent there are groups of children who specialise in given forms and functions of aggressive behaviours, either independently or simultaneously, as well as to what extent these specialisations change during the course of development. For example, in one study using a variable-centered approach to model both forms and functions of aggression trajectories in adolescence (i.e., from 12 to 14 years of age), Ojanen and Kiefer (2013) found that on average, there were increases in instrumental (i.e., proactive) relational aggression and decreases in reactive overt aggression across time. These findings are in line with previous studies of the individual and joint trajectories of aggression and shed important insights into a more holistic view of continuity and discontinuity, of form and functions of aggression in adolescence specifically. However, an important methodological consideration in interpretation of these findings was the inability to examine non-linear trajectories. As the authors acknowledge, linear models may not be best suited to modelling trajectories of forms and functions of aggression. Moreover, given the social-cognitive-behavioural changes occurring at entry into formal schooling, it would be highly informative to start examining these co-occurring trajectories of forms and functions of aggression at earlier stages, prior to adolescence. Particularly so given this important developmental period when the opportunities for social interactions with peers have substantially increased. Thus, one aim of our study was to extend upon the above findings by examining a longer developmental period, using a person-centered approach, whilst not restricting the shape of co-occurring forms and functions of aggression trajectories.

## Antecedent Risk Factors

Studies of individual and joint subtypes of aggressive behaviour have shown that persisting childhood aggression carries high social burden and economic cost to societies. To prevent these chronic trajectories of aggression, we need to understand to what extent different types of aggression feed into one another during development. Particularly so given that chronic engagement in multiple forms and functions of aggression likely carries even higher risk for consequent negative life outcomes. Conversely, if we can identify groups of children who predominantly exhibit one type of aggression only, then it becomes important to examine whether individuals exhibiting distinct types of aggression have different antecedent characteristics and risk factors associated with group membership. Given that this is the first study to the best of our knowledge, looking at multi-trajectories of both forms and functions of aggression within a developmental framework, whilst using a person-centered approach, we start by examining whether common antecedent factors previously identified in single trajectories of aggression, are also predictive of potential co-occurring trajectories. Indeed, different intervention strategies may be required for the prevention of different types of aggression and especially for different mixtures of aggression types. Thus, understanding whether there are distinct patterns of group membership for co-morbid engagement, and the antecedent characteristics associated with each group, is an important first step in the development of effective prevention strategies.

In a review of the literature, some key antecedent family-, maternal-, and child-level risk factors of both individual forms and functions of aggression were identified. For example, at the family level, lower level socio-economic status and family status (i.e., single mothers) have conferred increased risk for elevated physical and indirect aggression in offspring (Côté et al. 2006, 2007; Tremblay et al. 2004; Vaillancourt et al. 2007). Additionally, in line with the theoretical model proposed by Dodge (1991), the origins of reactive and proactive aggression stem from early social experiences and in particular parenting behaviours such as harsh and coercive parenting (Vitaro et al. 2006). Harsh and coercive types of parenting behaviours have also been implicated in engagement in both physical and indirect aggression (Campbell et al. 2010; Hentges et al. 2018; Orri et al. 2018; Girard et al. 2014; Tremblay et al. 2004; Côté et al. 2007; Vaillancourt et al. 2007). At the maternal level, risk factors such as education and IQ, age at birth of first child, smoking during pregnancy, previous antisocial history during adolescence, and depression, have all been implicated as risk factors for children who display with higher levels of aggression (e.g., Huijbregts et al. 2008; Tremblay et al. 2004; Côté et al. 2007; Hay et al. 2003). For a comprehensive overview of implicated family and maternal level risk factors related to chronic physical aggression specifically, see Tremblay et al. (2018). With respect to child-level factors, both receptive and expressive language (e.g., Dionne 2005), preterm birth (e.g., Potijk et al. 2012), low birth weight (e.g., Pharoah et al. 1994), sibling status (e.g., Stauffacher and DeHart 2006; Goodwin and Roscoe 1990), and sex (e.g., Tremblay et al. 2004), have all been found to increase the risk of higher engagement in subtypes of aggressive behaviours. Given previous identification of the above-mentioned risk factors for individual and/or joint forms and functions of aggression, we examined whether these same risk factors would predict membership in trajectories of combined forms and functions of aggression. Additionally, we also examined breastfeeding. There has been increasing interest in examining the association between breastfeeding and externalising behaviours such as conduct problems, which has yielded mixed results (e.g., Jackson 2016; Girard et al. 2017; Girard et al. 2018). Proposed mechanisms for the association include psychological (e.g., via attachment), brain development (e.g., via nutrients and white growth matter), and genetic risk. It may then be possible that breastfeeding is implicated in forms or functions of aggression.

## Objectives

The first objective was to examine the heterogeneity in developmental trajectories of combined physical, indirect, proactive, and reactive aggression across childhood and into adolescence. Rooted within a developmental model and the early childhood perspective of aggression, we expected that a majority of children would follow moderate to low decreasing physical aggression trajectories with variation in stable to increasing indirect aggression over time. We also predicted that these same children would likely decrease in reactive aggression over time, with potentially increasing proactive aggression in children who were following increasing trajectories of indirect aggression. Additionally, and in line with previous findings, we expected to find a small group of children who engaged in high and chronic forms and functions of aggression over time. Finally, we expected to see a group of children who did not engage in either forms or functions or aggression over time. Taken together, we expected to find a three or four group model to best fit the data.

The second objective was to better understand which antecedent characteristics, at the child, maternal, and family level, were associated with group membership. We made no predictions regarding the specific antecedent characteristics that would distinguish between differing trajectories given that this is the first study to examine multi-trajectories of both forms and functions of aggression across childhood and into adolescence. We did however expect to find that the previously identified characteristics outlined above would similarly be implicated in the multi-trajectory groups identified in the current study. Finally, we examined the risk associated with these antecedent characteristics for group membership, which distinguished the chronic groups from the others. Here we expected to find that children, particularly boys from lower SES backgrounds, who were from single parent families, with younger mothers who had a history of engagement in anti-social behaviour along with lower IQ or educational background, who also engaged in high-risk behaviours during pregnancy such as smoking, and who used harsh and coercive parenting practices, would be at higher risk of membership in the chronic group.

## Method

### Participants

This study uses data collected from children enrolled in the Quebec Longitudinal Study of Child Development (QLSCD). The QLSCD is a nationally representative cohort of singletons born in Quebec, Canada, between 1997 and 1998 that were selected from the Quebec birth registry. Sample selection and stratification procedures have been extensively documented (Jette and Des Groseilliers 2000). The initial sample was comprised of 2223 children and their families. Inclusion criterion in the current study was having a minimum of three assessments for each subtype of aggression, thus resulting in a final sample of 787 children. The inclusion criterion was required given that three data points are necessary to properly fit a quadratic polynomial term in the group-based multi-trajectory approach. The sample characteristics of the entire cohort as compared to those included in the current study can be found in Table 1. Informed written parental consent and children’s assent were collected prior to each wave of data collection. Ethical approval was obtained by the Québec Institute of Statistics’ Ethics Committee.

**Table 1:** Demographic characteristics of entire sample and families included in the current study

	Excluded Sample (*N* = 1436)	Current Sample (*N* = 787)	*p*
Highest Maternal Diploma:			<0.001
No Diploma	288 (20%)	113 (14%)	
High School Diploma	384 (27%)	200 (25%)	
College Diploma	413 (29%)	231 (29%)	
University Diploma	348 (24%)	243 (31%)	
Maternal Age:			0.742
Less than 20 years	45 (3%)	19 (2%)	
20–24 years	285 (20%)	145 (18%)	
25–29 years	454 (32%)	248 (32%)	
30–34 years	458 (32%)	271 (34%)	
35–39 years	164 (11%)	86 (11%)	
More than 40 years	29 (2%)	18 (2%)	
Maternal Working Status (Unemployed):	451 (32%)	203 (26%)	0.004
Maternal Smoking during Pregnancy (Yes):	373 (26%)	182 (23%)	0.332
Maternal Ethnicity (Non-Canadian):	566 (40%)	245 (31%)	<0.001
Maternal Antisocial Behaviour	0.9 (1.0)	0.7 (0.8)	0.002
Single Parent Family (Yes):	133 (9%)	41 (5%)	0.003
Family Income Less than 19,999 (Yes):	275 (20%)	83 (11%)	<0.001
Child Sex (Boys):	800 (56%)	338 (43%)	<0.001

Means and (standard deviations) are presented for maternal adolescent antisocial behaviour

### Outcome Measures

Teacher-reports were used to collect information on aggressive behaviours at six, seven, eight, 10, 12 and 13 years of age. The teacher-report used in the QLSCD cohort was taken from the Canadian National Longitudinal Survey of Children and Youth (NLSCY), which used items from the Child Behaviour Checklist (i.e., comprised of118 items assessing problem behaviours), the Children’s Behaviour Questionnaire (i.e., comprised of 195 items assessing temperament), and the Social Behaviour Questionnaire (i.e., comprised of 38 items assessing both problem and prosocial behaviours). All scales have previously been well validated in the literature (Achenbach and Edelbrock 1983; Rutter 1967; Tremblay et al. 1991). In the current study, we used only items measuring forms and functions of aggression. That is, teachers rated the frequency of children’s engagement in physical, indirect, proactive, and reactive aggression on a scale from *0* (*never*) to *3* (*always*). The numbers of items differed across subtype of aggression and thus all subscales were rescaled to range between 0 and 10. Physical aggression (PA) included three items (i.e., physically attacks others; fights often with others; hits, bites, kicks others). Cronbach’s alpha in the current sample at 6, 7, 8, 10, 12, and 13 years was 0.88, 0.86, 0.87, 0.89, 0.87, and 0.85, respectively. Indirect aggression (IA) included three items (i.e., when angry with someone, tries to get others to dislike that person; when angry with someone, became friends with another as revenge; when angry with someone, says bad things behind the other’s back). Cronbach’s alpha at 6, 7, 8, 10, 12, and 13 years was 0.85, 0.88, 0.87, 0.88, 0.86, and 0.90, respectively. Proactive aggression (PAA) also included three items (i.e., intimidates others to get what he/she wants; tries to dominate other children; encourages children to pick on a particular child). Cronbach’s alpha at 6, 7, 8, 10, 12, and 13 years was 0.74, 0.77, 0.72, 0.77, 0.83, and 0.84, respectively. Finally, reactive aggression (RA) included four items (i.e., reacts in an aggressive manner when something is taken away from him/her; reacts in an aggressive manner when contradicted; reacts in an aggressive manner when teased; when hurt by another child, gets angry and reacts by fighting). Cronbach’s alpha at 6, 7, 8, 10, 12, and 13 years was 0.89, 0.87, 0.86, 0.89, 0.85, and 0.92, respectively.

### Antecedent Characteristics

To better understand the etiology and specific risk factors associated with concomitant aggressive trajectories, maternal reports and standardised measures were used. When children were five months, mothers reported on whether having obtained a high school diploma (yes/no), maternal age at the birth of her first child (21 years or less/22 years or over), annual household income during the past 12 months (dichotomized into less than $19,999, over 20,000 Canadian dollars, representing low income families; Statistics Canada 2011), family status at birth (single parent/dual parent), maternal smoking during pregnancy (yes/no), maternal depression (a score of ≥16 using the Centre for Epidemiological Study of Depression, short version, CES-D; Radloff 1977), which has been previously well validated (Lewinsohn et al. 1997), type of delivery (vaginal/caesarean), preterm birth derived from gestational age (delivered prior to the start of the 37th week, yes/no), the child’s birth weight (≥2500 g, yes/no), and the child’s birth order. Maternal adolescent antisocial behaviour was assessed at five months using seven items previously validated in the literature (Zoccolillo et al. 2004). Examples of the items include: before you completed your secondary school studies had you already been involved with the direction of youth protection or with the police, or had you been stopped by the police because of your bad behavior. Cronbach’s alpha is 0.92. The Cumulative Score for Neonatal Risk (CSNR) comprised of the infant’s APGAR score, birth weight, gestational length, congenital abnormalities, retardation of cranial perimeter growth, retardation of intrauterine growth and neonatal complications was taken from medical records at birth. At one and a half years, mothers were asked about breastfeeding engagement (scored as never breastfed, up to six months, more than six months).

A proxy of maternal IQ (i.e., crystallized intelligence) was assessed when children were age five years, with 14 items measuring receptive ability. For each item, mothers were asked to fill in the missing word that most correctly completed the idea of the sentence from a list of five potential options. Positive and coercive parenting was collected using the Parent Practices Scale (Strayhorn and Weidman 1988) when children were one and a half (positive only), two and a half, three and a half, four and a half, and five years. Cronbach alpha for positive parenting was 0.64, 0.62, 0.62, 0.63, and 0.65, respectively, and 0.68, 0.71, 0.67, and 0.74, respectively for coercive parenting. A mean composite score over time was then created. The scale’s reliability and validity have been well documented (Strayhorn and Weidman 1988). The Peabody Picture Vocabulary Test (PPVT; Dunn and Dunn 1997), a standardized measure of children’s receptive language was also assessed when children were six years; Cronbach’s alpha, 0.93. The PPVT has demonstrated good reliability and validity (e.g., Campbell 1998).

### Statistical Analysis

There remains ongoing debate surrounding the use of residuals in modelling forms and functions of aggression given the challenges in conceptualising the residualised construct in a meaningful way, in addition to challenges with its validity (e.g., Miller and Lynam, 2006). Despite this, research in aggression must take into consideration the potential measurement and analytical challenges in examining both forms and functions as distinct subtypes of aggression (Little et al. 2003). Thus, we first ran a one, two, and four factor confirmatory factor analysis using the Mplus software, version 7.4. Model fit indices used to assess the best model fit, in addition to the Chi-square which is commonly significant in larger sample sizes, include the root mean square error of approximation (RMSEA), the comparative fit index (CFI), and the root mean square residual (SRMR). Good model fit can be represented by a RMSEA of equal to or less than 0.08 (MacCallum et al. 1996), a CFI of greater than 0.95 (Hu and Bentler 1999), and a SRMR of equal to or less than 0.05 (Diamantopoulos and Siguaw 2000). The model fits revealed that across all ages, the four-factor model continually provided the best fit to the data, suggesting discriminant validity. Model fit comparisons can be found in the online Supplement 1. Additionally, a correlation table between forms and functions of aggression across time is presented in Supplement 2.

Group-based modelling was conducted next. Unlike the traditional growth curve modelling approach (i.e., variable-centered) which focuses on population means, individual variability around the population mean, and the contributing factors for that variability, the group-based modelling approach (i.e., person-centered) aims to model distinctive trajectory groups within the population and to identify factors that differentiate the groups (Nagin 2005). It is a semi-parametric approach to modelling the heterogeneity of developmental trajectories within the targeted population. A recent extension of the group-based trajectory model, called the multi-trajectory approach, was used in the current study to model multiple subtypes of aggression jointly from childhood to adolescence (Nagin et al. 2016). The advantage of this extension is its ability to conjointly model multiple subtypes of behaviours, thus providing an overall behavioural ‘profile’ of, in our case, aggression across multiple dimensions.

A two-stage approach was used to identify the best fitting model. First, two, three, four, five and six group models were run to compare the Bayesian information criteria (BIC) of each model (Supplement 3). A larger BIC is indicative of a better fitting model (D’Unger et al. 1998; Nagin 2005). Both four, five, and six group models provided the best BIC fits. Next, polynomial terms were fitted in the four, five, and six-group model. Based on these two criteria, a five group multi-trajectory model provided the best fit to the data and was selected. Further, criteria for judging the adequacy of the selected, suggested in Nagin (2005) such as the average posterior probabilities of group membership by assigned trajectory group and odds of correct classification, supported the adequacy of the five-group model. Model fit criteria are presented in Table 2.

**Table 2:** Model fit criterion of the multi-trajectories of subtypes of aggression

Trajectory Group	N	Average Posterior Probability of Group Membership	Odds of Correct Classification
1	203 (26.6%)	81.7%	12.8
2	272 (33.7%)	90.5%	17.9
3	56 (7.4%)	87.0%	87.1
4	218 (27.4%)	86.8%	17.1
5	38 (4.9%)	95.3%	402.8

Membership probability greater than 70 and OCC greater than 5 represent good model fit

Chi-square tests and analysis of variance were conducted to first understand which characteristics were significantly associated with group-membership. Next, multinomial logistic regression models were examined to better understand risk factors for belonging to the moderate-desisting, high-desisting, and chronic group as compared to the low to non-aggressive groups. Only factors that were statistically significant in bivariate analysis were examined in the multivariate model based on the principle of parsimony. The statistical threshold was set at *p* = 0.050, two-tailed. These analyses were performed using Stata 14.0 software. We use the term significant to denote statistical significance henceforth.

## Results

**Fig. 1:**
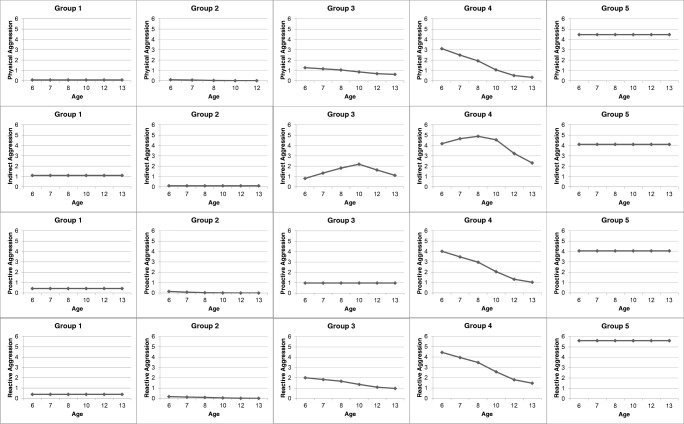
Multi-Trajectories of aggression subtypes from childhood to adolescence

Five groups of children emerged, displaying distinct developmental patterns of concomitant PA-IA-PAA-RA. The first group, which was estimated to compose 26.6% of the sampled population and labelled as the ‘low-stable’ group, were low engagers in PA, PAA, & RA. Levels of IA were slightly higher as compared to the other subtypes of aggression yet were still low across the developmental period from six to 13 years of age. This group was entirely stable in their engagement in each subtype of aggression across time. The second, and largest group was estimated to compose of 33.7% of the sampled population. This group, labelled as the ‘non-aggressors’, were rated as not having engaged in any subtype of aggression between six and 13 years of age. The third group, the ‘moderate-engaging’ group, were rated as having moderate engagement in each subtype of aggression at age six, followed by a linear decreasing trajectory of PA, and RA thereafter. The frequency of IA engagement in this group revealed a quadratic rather than linear shape, whereby IA was moderate at age six, increased between seven to 10 years, and gradually returned to moderate engagement between 10 and 13 years. The shape of engagement in PAA in this group however did not decrease over time, but rather it remained stable. The moderate-engaging group was estimated to account for 27.4% of the sampled population. Group four, the ‘high-desisting’ group revealed similar shapes in their trajectories of PA, IA, and RA as the moderate-engaging group, albeit at higher levels of initial aggression and throughout development. Further, PAA in this group followed a linear decreasing trajectory rather than remaining stable as in the case of the moderate-engaging group. This group was estimated to compose 7.4% of the sampled population. The fifth and final group, consisted of an estimated 4.9% of the sampled population, and were labelled as the ‘high-chronic’ engagers. This group consistently engaged in elevated and stable levels of all forms aggression from age six to 13. Multi-trajectory groups are displayed in Fig. 1.

Based on chi-square tests and analysis of variance, Table 3 displays antecedent child, maternal, and family characteristics associated with group membership. At the child level, sex was the only significant characteristic. At the maternal level, highest diploma obtained, maternal IQ, maternal adolescent antisocial behaviour, and postnatal depression, were all significant. Family characteristics were not found to distinguish between group-membership although parenting factors such as breastfeeding and coercive parenting were.

**Table 3:** Proportions and mean level of risk factors for the five distinct multi-aggression trajectory groups: bivariate analysis

	Low Stable (*n* = 203)	Non-Aggressors (*n* = 272)	Moderate Engagers (*n* = 218)	High Desisting (*n* = 56)	High Chronic (*n* = 38)	*p*
Family Factors						
Low income (less than $19,999 annually):	6.5%	11.0%	11.1%	14.3%	21.1%	0.063
Single parent family:	5.4%	3.3%	5.5%	7.1%	13.2%	0.120
Maternal Factors						
Less than high school diploma:	11.8%	11.4%	15.1%	23.2%	31.6%	0.003
Less than 21 years at birth of 1st child:	18.0%	16.1%	21.8%	20.0%	31.6%	0.163
Smoking during pregnancy:	22.2%	20.6%	24.8%	23.2%	36.8%	0.103
Postnatal depression:	9.9%	8.1%	12.8%	26.8%	23.7%	<0.001
Child Factors:						
Born preterm:	6.4%	2.6%	7.3%	3.6%	2.6%	0.113
Low birth weight:	5.9%	2.6%	3.7%	1.8%	0.0%	0.202
Caesarean birth:	14.8%	12.9%	18.1%	10.7%	10.5%	0.416
First born child:	42.9%	49.3%	44.0%	37.5%	50.0%	0.397
Sex: Boy	24.1%	37.1%	61.0%	41.1%	84.2%	<0.001
Parenting Factors:						
Never breastfed:	22.2%	21.5%	32.1%	33.9%	43.2%	<0.001
Means						
Maternal adolescent antisocial behaviour:	0.8 (0.9)	0.6 (0.8)	0.8 (0.8)	1.0 (0.9)	0.8 (0.8)	0.037
Maternal IQ:	8.3 (0.9)	8.2 (1.0)	8.2 (0.9)	7.9 (1.0)	7.5 (1.2)	<0.001
Positive Parenting:	6.8 (0.7)	6.8 (0.9)	6.8 (0.9)	6.7 (0.8)	6.7 (0.6)	0.972
Coercive Parenting:	2.3 (0.7)	2.2 (0.7)	2.5 (0.8)	2.5 (0.6)	2.7 (0.8)	<0.001
Receptive Language (child at 6 years):	117.2(15.0)	117.7(17.1)	117.2(15.3)	113.5(14.5)	113.4(17.2)	0.333
Risk at birth (CSNR):	0.9 (1.1)	0.9 (1.1)	1.0 (1.3)	0.8 (1.2)	0.9 (1.2)	0.209

Maternal IQ is scored on a scale from 0 to 10 with higher scores indicative of higher IQ. Percentages of prevalence of antecedent characteristic per group displayed for chi-square analysis; means and (standard deviations) presented in analysis of variance

Finally, to better understand which characteristics were associated with risk for belonging to trajectory groups, a multinomial logistic regression was conducted by grouping the low-stable and non-aggressors together to form the reference group, and then comparing the moderate-engaging, high-desisting, and high-chronic groups; Model *x*^2^ = 151.62, *p* = <0.001, pseudo *R*^2^ = 0.12. For the moderate-engaging group, coercive parenting, never being breastfed, and being male were all risk factors that increased the relative risk ratio of membership in this group. Maternal and parental risk factors that increased the relative risk ratio of membership in the high-desisting group included lower maternal IQ, and postnatal depression. Finally, maternal risk factors for membership in the high-chronic group included maternal education and IQ. Coercive parenting also increased the relative risk ratio for high-chronic aggression. The strongest risk factor for group membership in the high-chronic group was being male. Please see Table 4.

**Table 4:** Risk factors associated with multi-aggression trajectories: multivariate analysis

	Moderate-Engagers	High-Desisting	High-Chronic
Maternal Diploma (Less than High School):	1.5(0.4) [0.8–2.8]	1.9(0.8) [0.8–4.3]	4.3(2.3) [1.5–12.4]
Maternal IQ: (High)	1.0(0.1) [0.8–1.2]	0.7(0.1) [0.5–0.9]	0.6(0.1) [0.4–0.9]
Maternal Postnatal Depression (Yes):	1.6(0.5) [0.8–3.1]	3.2(1.3) [1.4–7.4]	2.9(1.5) [0.9–8.3]
Maternal Adolescent Antisocial Behaviour:	1.1(0.1) [0.8–1.3]	1.3(0.2) [0.8–1.7]	1.1(0.2) [0.6–1.7]
Coercive Parenting:	1.7(0.2) [1.2–2.1]	1.5(0.3) [0.9–2.2]	2.5(0.6) [1.4–4.2]
Breastfeeding			
Never:	2.2(0.5) [1.2–3.6]	2.2(1.0) [0.8–5.7]	2.3(1.3) [0.7–7.2]
Up to 6 months:	1.2(0.2) [0.7–1.9]	2.1(0.8) [0.8–4.7]	0.9(0.5) [0.3–2.9]
Child Sex (Boy):	3.9(0.7) [2.6–5.8]	1.8(0.5) [0.9–3.4]	13.5(7.7) [4.4–41.3]

The comparison group combined the low-stable and non-aggressors. The reference category for breastfeeding was six months or more. The relative risk ratio is presented along with the (standard error) and the [95% Confidence Intervals]

## Discussion

This is the first study which has used the multi-trajectory approach to be able to model both forms and functions of aggression simultaneously across development. In so doing, allowing us to visually represent distinct groups of children who exhibit unique patterns of both change and continuity over time, in and across, concurrent forms and functions of aggression. Our overarching aims, were to identify any heterogeneity in the co-occurrence of PA, IA, PAA, and RA using this group-based multi-trajectory approach, and to identify risk factors, specific to the unique trajectories identified, that may be good targets for early prevention and/or intervention efforts. These questions are of particular importance given the multitude of emergent maladaptive outcomes experienced over the life course for persistent aggressors.

The literature has typically placed the examination of subtypes of aggression within a single or dual-dimensional context only, whereby a focus on either form (PA-IA) or functions (PAA-RA) are studied in combination, with two-, three- and four group trajectory models often being identified (e.g., Barker et al. 2006; Côté et al. 2007; Nagin and Tremblay 1999). Our findings, while in line with previous developmental patterns found in these studies (e.g., groups of non-engagers, high-desisting, chronic engagers), offer new insights on observable patterns of four co-occurring subtypes of aggression from six to 13 years of age. Our results suggest that both subtype and severity models are necessary for early programming efforts given the differing trajectories. Moreover, the differences in antecedent factors identified between these five trajectory groups has important implications for developmental models of aggression as the field moves forward, given that there was little overlap between the risk factors implicated across group membership in groups displaying with moderate to chronic levels of aggression.

### Heterogeneity in Multi-Trajectories of Aggression and Theoretical Perspectives

Our first hypothesis, based on previous work of single and joint trajectories, was that a three- or four-group model would best fit the data. Our data however best supported a five-group model: low-stable, non-aggressors, moderate-engaging, high-desisting, and high-chronic. We further hypothesised that a majority of children would engage in moderate to low decreasing PA with variation in stable to increasing IA over time, coupled with decreases in RA over time, and potentially increasing PAA for children who were following increasing trajectories of IA. Additionally, we expected to find a small group of children who engaged in high-chronic forms and functions of aggression over time along with another group of children not engaging in either forms or functions of aggression over time. Our data partially supported this hypothesis. From the age of six to 13 years, 60.3% of children were rarely engaging in any subtype of aggression, 34.8% engaged in some form of mostly desisting trajectory and only 4.9% remained stable in their use of elevated subtypes of aggressive behaviours. However, given the host of associated maladaptive outcomes across the life course for chronic engagers, the 4.9% who persist in both form and function of aggression, is not negligible.

From a theoretical perspective, the findings in our study (for children in trajectory groups who were engaging in aggressive behaviours), appear most strongly aligned with the early childhood perspective of aggression. For example, most children who engage in aggressive behaviours are learning how *not* to aggress as they grow older (Nagin and Tremblay 1999; Tremblay et al. 1999; Tremblay 2010), rather than learning to aggress with time via modelling and conditioning, as is suggested in the social learning theory of aggression (Bandura 1973). Support for this early childhood perspective is demonstrated through the decreases found in aggressive trajectories from childhood to adolescence in the multi-trajectory groups identified in our study. The findings not only support this perspective for physically aggressive behaviours, but it appears that when modelling concomitant forms and functions of aggression, this perspective also fits with reactive, and to some extent, proactive aggression (i.e., group 4). Indirect aggression on the other hand revealed slight digressions from this early unlearning perspective, in particular during middle childhood.

That is, both the moderate-engaging and high-desisting groups revealed an interesting pattern with respect to their engagement in indirect forms of aggression. While PA and RA were linearly decreasing, IA increased between seven and 10 years before eventually also decreasing. This suggests that while these children are learning how not to engage in overt and reactive displays of aggression, they are also learning more sophisticated forms of covert aggression, perhaps to replace the less socially accepted forms of PA and RA as they grow older, at least in the interim and prior to adolescence (Côté et al. 2007). Indirect aggression has been argued to coincide with the development of linguistic competence and social intelligence (Björkqvist et al. 1992b, 2000; Garandeau and Cillessen 2006), which may approximate the reason for the observed peak at a later developmental stage. However, the steady decline of IA in both groups between 10 years of age and adolescence suggest that a developmental trajectory of inhibiting the frequency of engagement in aggressive behaviours over time may also apply to indirect forms of aggression just prior to adolescence. The period between 10 and 13 years of age for this sample marks the transition between elementary school and high-school. Thus, an alternative explanation for the observed decrease in IA after age 10 may be reflective of this transition period. While children are starting to increase their use of indirect aggression, the transition may help to suppress its continuation with shifts in positions within school-based social hierarchies.

Regarding the high-chronic group of aggressors, (i.e., mainly boys), our results revealed that there were no preferences regarding either forms or functions of aggression used. Given the stability of engagement in multiple subtypes of elevated aggression from kindergarten onwards, this group of children appear at greater risk for continued aggression, mental health problems and maladaptive functioning in later adolescence and adulthood.

### Antecedent Characteristics Associated with Group-Membership

As this is the first study to the best of our knowledge to examine multi-trajectories of both forms and functions of aggression trajectories across an eight-year developmental period, we made no hypotheses with respect to the specific risk factors that would predict group membership across the individual trajectories identified. We did however expect to find that for any chronic group identified, risk factors would include being male, from young single-mother families, with their own history of engagement in anti-social behaviour, lower IQ, educational, and SES backgrounds, and who engaged in high-risk prenatal behaviours (i.e., smoking). Further, we expected higher levels of coercive parenting behaviours to predict membership in the chronic group. Partial support for this hypothesis was found and is discussed below.

We identified specific risk and protective factors that were both homogeneous and heterogeneous between the moderate-engagers, high-desisting and high-chronic groups as compared to the combined low-stable and non-aggressor groups, although the majority of factors in combination were specific to individual trajectory groups. One factor, being male, was a risk factor common to two trajectory groups (i.e., the moderate-engagers and high-chronic), although this risk was largest for the high-chronic group (i.e., a staggering relative risk ratio of 13.5). This finding is line with previous studies examining either forms or functions of aggression and suggests that young males engaging in high levels of early aggression may need additional programming support to provide them with alternative strategies to replace their use of aggressive behaviours.

Another common risk factor was maternal IQ. That is, lower maternal IQ was a a risk factor associated with membership in both the high-desisting and high-chronic groups in particular. More specifically, for every decreasing unit (i.e., point scored), the relative risk ratio increased by 30 and 40%, respectively. While another factor (i.e., coercive parenting), was also common to two groups (i.e., the moderate-engagers and the high-chronic groups), albeit with differing magnitudes. For example, coercive parenting was associated with higher probabilities of membership in the high-chronic group as compared to the moderate-engagers, which would be expected. Coercive parenting, has repeatedly been implicated in children’s aggressive behaviour, which is likely attributable to both genetic transmission of risk and negative early child-rearing environments (reflected in poor quality interactions between the mother and child; e.g., Tremblay et al. 2004). Thus, the implication of coercive parenting for membership in the moderate-engagers and high-chronic trajectories would suggest that inadequate parenting skills are a good target for early prevention efforts. It was unclear however why coercive parenting was not associated with higher probabilities of membership in the high-desisting group, warranting future investigation.

Heterogeneous risk factors were also identified. For example, mothers not having received a high school diploma increased the relative risk ratio for membership in the high-chronic group by 4.3. This finding is in line with Nagin and Tremblay (2001) who also found low maternal education to increase risk for being on a chronic trajectory of PA. Moreover, maternal postnatal depression had an impact on membership in the high-desisting group: a relative risk ratio of 3.2. Maternal depression has previously been implicated in children’s aggressive behaviour (e.g., Hay et al. 2003), and is likely implicated via similar pathways as that of coercive parenting, particularly regarding early poor-quality dyadic interaction (e.g., Kim-Cohen et al. 2005). Interestingly, not being breastfed appeared to increase the risk for membership in the moderate-engagers trajectory group. This finding provides some support to the growing body of work examining the association between breastfeeding and externalising problems. While it is not entirely clear why not being breastfed would only be implicated for the moderate-engaging group, this finding is consistent with the suggestion that breastfeeding may only be associated with externalising problems at non-clinical levels (Girard et al. 2017).

Taken together, these findings would suggest that early intervention efforts really ought to be tailored dependent on group membership, as risk factors, and the combination of risk factors, are not uniform across concomitant trajectories of form and function of aggression engagement. Moreover, our results highlight the need for additional studies that use person-centered, rather than variable-centered approaches to modelling aggression across development. While the popular variable-centered approach in developmental studies has critically advanced theory in developmental aggression, it may have also partially inhibited our understanding of the degree of heterogeneity among groups of children engaging in concomitant forms and functions of aggression, along with the antecedent characteristics that increase risk for specific group membership.

### Strengths and Limitations

As this is the first study to simultaneously model both forms and functions of aggression and to estimate the ways in which they feed into one another using a developmental multi-trajectory person-centered approach, there are notable strengths. These include the use of a semi-parametric approach for modelling heterogeneity in four distinct subtypes of aggression simultaneously, the relatively large sample size, the use of repeated measures across time and assessment of maternal IQ, along with the use of a multi-informant approach whereby limiting the potential of shared method variance. Despite these strengthens, the study has limitations that must be acknowledged. Good practice for properly fitting a polynomial term requires a minimum of three data points per child, which significantly reduced the sample size. Thus, our results may be specific to the subsample used in this study, despite being largely consistent with previous epidemiological findings. Further, statistically significant differences were found between the entire cohort and those included in the current study, two of which were found to be significant risk factors for group membership in the high-chronic group (i.e., maternal education and child sex). Thus, it is possible that the prevalence rate of those belonging to the high-chronic group may in fact be underrepresented, warranting replication with larger sample sizes. Additional studies are also needed to examine a longer period of development (i.e., from infancy into adulthood), and which evaluate the long-term outcomes associated with specific group membership. The internal consistency for parenting variables were also low, which may have resulted in underestimation of the associations, thus warranting replication in future studies. Moreover, as with any study examining aggression, concerns may be raised with either construct validity or reliability across forms and functions of aggression variables. As a result of the challenges in providing a meaningful conceptualisation of residualised constructs when modelling forms and functions of aggression, we did not use residualised constructs in the current study. Thus, the possibility of confounding remains, particularly with respect to items for indirect aggression which may have also captured elements of reactive aggression. The results of the confirmatory factor analysis however do provide support for the construct validity of aggression variables used. Finally, additional items measuring both forms and functions of aggression would have been desirable. The high Cronbach’s alphas provided in this study however, provide support for the reliability of aggression variables used.

### Conclusions

Our results are in line with previous studies suggesting that programmes for the prevention of aggression should be offered to mothers with lower levels education or cognitive capacities (Eckenrode et al. 2017; Enoch et al. 2016). Further, children of mothers with a history of depression or who may have a higher disposition for postnatal depression may benefit from preventative efforts. These mothers in particular may need additional supports for strategies to provide their children with rich and stimulating environments from pregnancy throughout childhood (Enoch et al. 2016). Such programming efforts ought to focus on teaching parents the strategies needed for promoting positive quality dyadic interactions along with disciplinary tactics that are not coercive in nature. Additional support should also be given for mothers of young boys.

## Electronic supplementary material

ESM 1 (DOCX 15.7 kb)

ESM 2 (DOCX 23.5 kb)

ESM 3 (DOCX 13.6 kb)
