# Supplementary material for: Development of Aggression Subtypes from Childhood to Adolescence: a Group-Based Multi-Trajectory Modelling Perspective
Source: J Abnorm Child Psychol. 2018 Nov 7;47(5):825–38. doi: 10.1007/s10802-018-0488-5 (PMC6469854; doi:10.1007/s10802-018-0488-5)
Supplement: Supplementary file 1 — (DOCX 15.7 kb) [file 10802_2018_488_MOESM1_ESM.docx]

Supplement 1: Confirmatory Factor Analysis of Aggression Forms and Functions - Model Fit Comparisons

|  | One Factor Model | | | | Two Factor Model | | | | Four Factor Model | | | |
| --- | --- | --- | --- | --- | --- | --- | --- | --- | --- | --- | --- | --- |
|  | χ2(*p*) | RMSEA (C.I) | CFI | SRMR | χ2 (*p*) | RMSEA (C.I) | CFI | SRMR | χ2 (*p*) | RMSEA | CFI | SRMR |
| Age 6 | 1295.77 (*) | .173 (.165- .181) | .749 | .106 | 606.03 (*) | .116 (.107-.124) | .889 | .073 | 259.71 (*) | .073 (.064- .083) | .959 | .041 |
| Age 7 | 1398.36 (*) | .165 (.158- .173) | .763 | .093 | 612.80 (*) | .107 (.099- .115) | .902 | .059 | 302.129 (*) | .074 (.066- .083) | .957 | .038 |
| Age 8 | 1584.982 (*) | .180 (.172- .188) | .676 | .111 | 774.351 (*) | .124 (.116-.132) | .849 | .072 | 306.215 (*) | .076 (.068- .085) | .947 | .042 |
| Age 10 | 1345.65 (*) | .173 (.165- .181) | .764 | .092 | 748.47 (*) | .127 (.119- .136) | .874 | .069 | 250.75 (*) | .070 (.061- .079) | .965 | .032 |
| Age 12 | 1340.29 (*) | .174 (.166- .182) | .725 | .104 | 611.27 (*) | .115 (.107- .123) | .882 | .071 | 253.30 (*) | .071 (.062- .080) | .958 | .045 |
| Age 13 | 1173.77 (*) | .162 (.154- .170) | .814 | .080 | 783.05 (*) | .131 (.123- .140) | .879 | .072 | 571.34 (*) | .115 (.107- .124) | .914 | .057 |

Note: (*) denotes statistical significance at the *p* = <.000 level.
