# Supplementary material for: Development of Aggression Subtypes from Childhood to Adolescence: a Group-Based Multi-Trajectory Modelling Perspective
Source: J Abnorm Child Psychol. 2018 Nov 7;47(5):825–38. doi: 10.1007/s10802-018-0488-5 (PMC6469854; doi:10.1007/s10802-018-0488-5)
Supplement: Supplementary file 2 — (DOCX 23.5 kb) [file 10802_2018_488_MOESM2_ESM.docx]

Supplement 2: Correlations between Forms and Functions of Aggression

|  | Physical_6 | Indirect_6 | Proactive_6 | Reactive_6 | Physical_7 | Indirect_7 | Proactive_7 | Reactive_7 | Physical_8 | Indirect_8 | Proactive_8 | Reactive_8 | Physical_10 | Indirect_10 | Proactive_10 | Reactive_10 | Physical_12 | Indirect_12 | Proactive_12 | Reactive_12 | Physical_13 | Indirect_13 | Proactive_13 | Reactive_13 |
| --- | --- | --- | --- | --- | --- | --- | --- | --- | --- | --- | --- | --- | --- | --- | --- | --- | --- | --- | --- | --- | --- | --- | --- | --- |
| PA_6 | 1 | .165 | .429** | .590** | .240* | .017 | .072 | .414** | -.011 | -.003 | .021 | .275** | .301** | .426** | .256** | .440** | .295** | .119 | .163 | .420** | .420** | .297** | .207* | .436** |
| IA_6 |  | 1 | .688** | .391** | .154 | .385** | .381** | .207* | .000 | .161 | .230* | .028 | .227* | .282** | .239* | .174 | -.052 | .189* | .195* | .127 | .005 | .079 | .115 | -.042 |
| PAA_6 |  |  | 1 | .510** | .241* | .192* | .272** | .317** | .021 | .152 | .170 | .000 | .317** | .302** | .251** | .292** | .106 | .226* | .283** | .299** | .213* | .227* | .269** | .174 |
| RA_6 |  |  |  | 1 | .303** | .256** | .286** | .584** | .072 | .208* | .217* | .292** | -.026 | .393** | .166 | .366** | .193* | .213* | .230* | .314** | .373** | .545** | .337** | .435** |
| PA_7 |  |  |  |  | 1 | .278** | .558** | .709** | .465** | .212* | .357** | .453** | .103 | .023 | -.007 | .518** | .482** | .345** | .536** | .522** | .246** | .211* | .178 | .165 |
| IA_7 |  |  |  |  |  | 1 | .711** | .307** | .169 | .491** | .420** | .163 | .007 | .140 | .302** | .144 | .134 | .263** | .339** | .146 | .076 | .281** | .141 | .125 |
| PAA_7 |  |  |  |  |  |  | 1 | .504** | .333** | .587** | .617** | .345** | -.030 | .132 | .236* | .264** | .285** | .338** | .443** | .346** | .147 | .273** | .200* | .094 |
| RA_7 |  |  |  |  |  |  |  | 1 | .382** | .221* | .313** | .495** | -.037 | .241* | .128 | .524** | .435** | .266** | .395** | .533** | .418** | .464** | .374** | .380** |
| PA_8 |  |  |  |  |  |  |  |  | 1 | .231* | .260** | .449** | .065 | -.067 | -.048 | .221* | .219* | .195* | .259** | .325** | .189* | .148 | .238* | .140 |
| IA_8 |  |  |  |  |  |  |  |  |  | 1 | .628** | .384** | -.069 | .143 | .191* | .119 | .104 | .415** | .331** | .312** | .124 | .249** | .085 | .162 |
| PAA_8 |  |  |  |  |  |  |  |  |  |  | 1 | .273** | -.067 | .065 | .102 | .201* | .207* | .409** | .424** | .379** | .072 | .139 | .044 | .041 |
| RA_8 |  |  |  |  |  |  |  |  |  |  |  | 1 | -.005 | .116 | -.039 | .432** | .460** | .256** | .297** | .597** | .240* | .236* | .014 | .225* |
| PA_10 |  |  |  |  |  |  |  |  |  |  |  |  | 1 | .132 | .346** | .278** | .024 | .114 | .131 | .116 | -.067 | -.078 | -.072 | -.066 |
| IA_10 |  |  |  |  |  |  |  |  |  |  |  |  |  | 1 | .585** | .339** | -.044 | .025 | .072 | .035 | .138 | .076 | .058 | .157 |
| PAA_10 |  |  |  |  |  |  |  |  |  |  |  |  |  |  | 1 | .203* | .081 | .069 | .116 | .022 | .193* | .140 | .144 | .199* |
| RA-10 |  |  |  |  |  |  |  |  |  |  |  |  |  |  |  | 1 | .383** | .292** | .393** | .507** | .291** | .252** | .169 | .301** |
| PA_12 |  |  |  |  |  |  |  |  |  |  |  |  |  |  |  |  | 1 | .325** | .497** | .599** | .518** | .327** | .232* | .410** |
| IA_12 |  |  |  |  |  |  |  |  |  |  |  |  |  |  |  |  |  | 1 | .746** | .503** | .281** | .397** | .273** | .325** |
| PAA_12 |  |  |  |  |  |  |  |  |  |  |  |  |  |  |  |  |  |  | 1 | .581** | .314** | .392** | .299** | .360** |
| RA_12 |  |  |  |  |  |  |  |  |  |  |  |  |  |  |  |  |  |  |  | 1 | .413** | .297** | .163 | .361** |
| PA_13 |  |  |  |  |  |  |  |  |  |  |  |  |  |  |  |  |  |  |  |  | 1 | .679** | .726** | .914** |
| IA_13 |  |  |  |  |  |  |  |  |  |  |  |  |  |  |  |  |  |  |  |  |  | 1 | .782** | .790** |
| PAA_13 |  |  |  |  |  |  |  |  |  |  |  |  |  |  |  |  |  |  |  |  |  |  | 1 | .750** |
| RA_13 |  |  |  |  |  |  |  |  |  |  |  |  |  |  |  |  |  |  |  |  |  |  |  | 1 |
