# Supplementary material for: Development of Aggression Subtypes from Childhood to Adolescence: a Group-Based Multi-Trajectory Modelling Perspective
Source: J Abnorm Child Psychol. 2018 Nov 7;47(5):825–38. doi: 10.1007/s10802-018-0488-5 (PMC6469854; doi:10.1007/s10802-018-0488-5)
Supplement: Supplementary file 3 — (DOCX 13.6 kb) [file 10802_2018_488_MOESM3_ESM.docx]

**Supplement 3: Comparison of the Bayesian Information Criteria (BIC) for Assessment of Model Fit**

|  | BIC | BIC | AIC |
| --- | --- | --- | --- |
| 2-group | -16149.70 (N=15519) | -16106.46 (N=787) | -16038.77 |
| 3-group | -15856.57 (N=15519) | -15793.95 (N=787) | -15695.92 |
| 4-group | **-15808.77 (N=15519)** | **-15726.78 (N=787)** | **-15598.40** |
| 5-group | **-15774.47 (N=15519)** | **-15673.10 (N=787)** | **-15514.38** |
| 6-group | **-15759.78 (N=15519)** | **-15639.03 (N=787)** | **-15449.97** |
